# Prepectoral implant-based breast reconstruction: a joint consensus guide from UK, European and USA breast and plastic reconstructive surgeons

**DOI:** 10.3332/ecancer.2019.927

**Published:** 2019-05-07

**Authors:** Raghavan Vidya, Giorgio Berna, Hani Sbitany, Maurice Nahabedian, Hilton Becker, Roland Reitsamer, Alberto Rancati, Douglas Macmillan, Simon Cawthorn

**Affiliations:** 1 Royal Wolverhampton Hospital, Wolverhampton WV10 0QP, UK; 2 Department of Plastic and Reconstructive Surgery, Ulss 9, General Hospital, Treviso, Italy; 3 Division of Plastic and Reconstructive Surgery, Department of Surgery, Mt Sinai Medical Center, New York, NY 10029 USA; 4 VCU College of Medicine—Inova Branch, National Center for Plastic Surgery, McLean, VA 22102 USA; 5 Voluntary Faculty Cleveland Clinic Florida Department of Plastic Surgery, Florida Atlantic University, Boca Raton, FL 33431 USA; 6 University Hospital Salzburg, Breast Centre Salzburg, Paracelsus Medical University Salzburg, 5020 Salzburg, Austria; 7 Henry Moore Oncologic Institute, Universidad de Buenos Aires, Buenos Aires C1053, Argentina; 8 Nottingham Breast Institute, Nottingham NG5 1PB, UK; 9 Bristol Royal Infirmary, Bristol BS2 8HW, UK

**Keywords:** breast, implant, reconstruction, prepectoral, ADM, mesh

## Abstract

Advances in implantable biologic and synthetic products over the last decade have enabled surgeons to replace traditional submuscular implant-based breast reconstruction techniques with a prepectoral or muscle-sparing technique. Prepectoral breast reconstruction is becoming increasingly popular among surgeons and patients due to the preservation of normal chest wall anatomy, with the restoration of body image with minimal morbidity. In this article, we have described a guide to prepectoral or muscle-sparing breast reconstruction with a particular emphasis on patient selection, technique and postoperative outcomes. Hence, a joint consensus guide from European and USA breast and plastic reconstructive surgeons has been agreed, and a crowd-writing method has been adopted to produce this guide.

## Recommendations

Recommendations have been derived after a review of published data and experience regarding the use of meshes in breast reconstruction. This document has been produced with the involvement of the breast and plastic reconstructive surgeons from the UK, Europe and the USA. The method of crowd-writing has been adopted. It is based mainly on available evidence, knowledge and clinical experience of the relevant experts to make these recommendations.

## Background

The use of implant-based reconstruction is increasing in the UK, Europe and the USA [1, 2]. This is due to the emergence of new implantable biologic and synthetic materials, as well as advancements in mastectomy and reconstruction techniques.

Prepectoral breast reconstruction has gained popularity in recent times as it avoids the surgical morbidity associated with chest wall muscle dissection; eliminating animation deformity and replacing the new breast implant in its normal anatomical plane where the breast tissue was removed [3].

However, prospective longitudinal data are scarce with limited reports on long-term postoperative outcomes; little guidance is available to aid with patient selection and the technical approach. As such, the goals of this consensus guide are:

- to define clinical and quality criteria for the prepectoral technique, including patient selection and postoperative outcomes.
- to suggest a standardised technical approach for the prepectoral-based technique.

## Methods

The panel of experts reviewed the literature and, in conjunction with their own experience, evaluated and graded the evidence to produce consensus recommendations and guidelines.

The panel was organised by Dr Raghavan Vidya and consisted of nine consultant surgeons (three oncoplastic breast surgeons; six plastic surgeons). All of the surgeons are involved in conducting multicentre studies on the prepectoral breast reconstruction technique and in performing and teaching it. Each section was written by one primary author and reviewed by all other authors. None of the authors reported any conflicts of interest. The panel’s discussions were conducted face-to-face and with the aid of electronic video conference calls, emails and telephone conference calls.

The scope of work consisted of technical considerations, patient selection criteria and evaluation on postoperative outcomes.

## Anatomy

Prepectoral space is the potential space that arises following the removal of the breast [4]. The volume of the space depends on the dimensions of the chest wall, along with the elasticity and quality of the skin, which dictates the degree to which it can safely accommodate the implant/mesh coverage.

## Indications

The main indication for this technique is immediate breast reconstruction following mastectomy for cancer or for risk-reducing surgery. It is also very useful in breast revision surgery, particularly to correct animation deformity and capsular contracture (Table 1).

## Clinic consultation

- Patients must be aware that they are being offered a relatively new procedure
- Patients must be completely informed about the procedure, advantages and disadvantages of implants, dermal matrices and meshes and other available alternatives.
- Patients must understand and accept that the reconstruction involves a breast implant for which there is no set lifespan although there is a likelihood for future revision for reasons such as capsule formation, asymmetry, implant visibility and palpability, implant rotation, implant rupture, infection and pain.
- Patients must be informed of the risk of anaplastic large cell lymphoma (ALCL) with textured implants (1 in 28,000).
- Patient must be informed about rippling and the options of corrective surgery (particularly lipomodelling).

## Suitable patients

**Figure 1.**
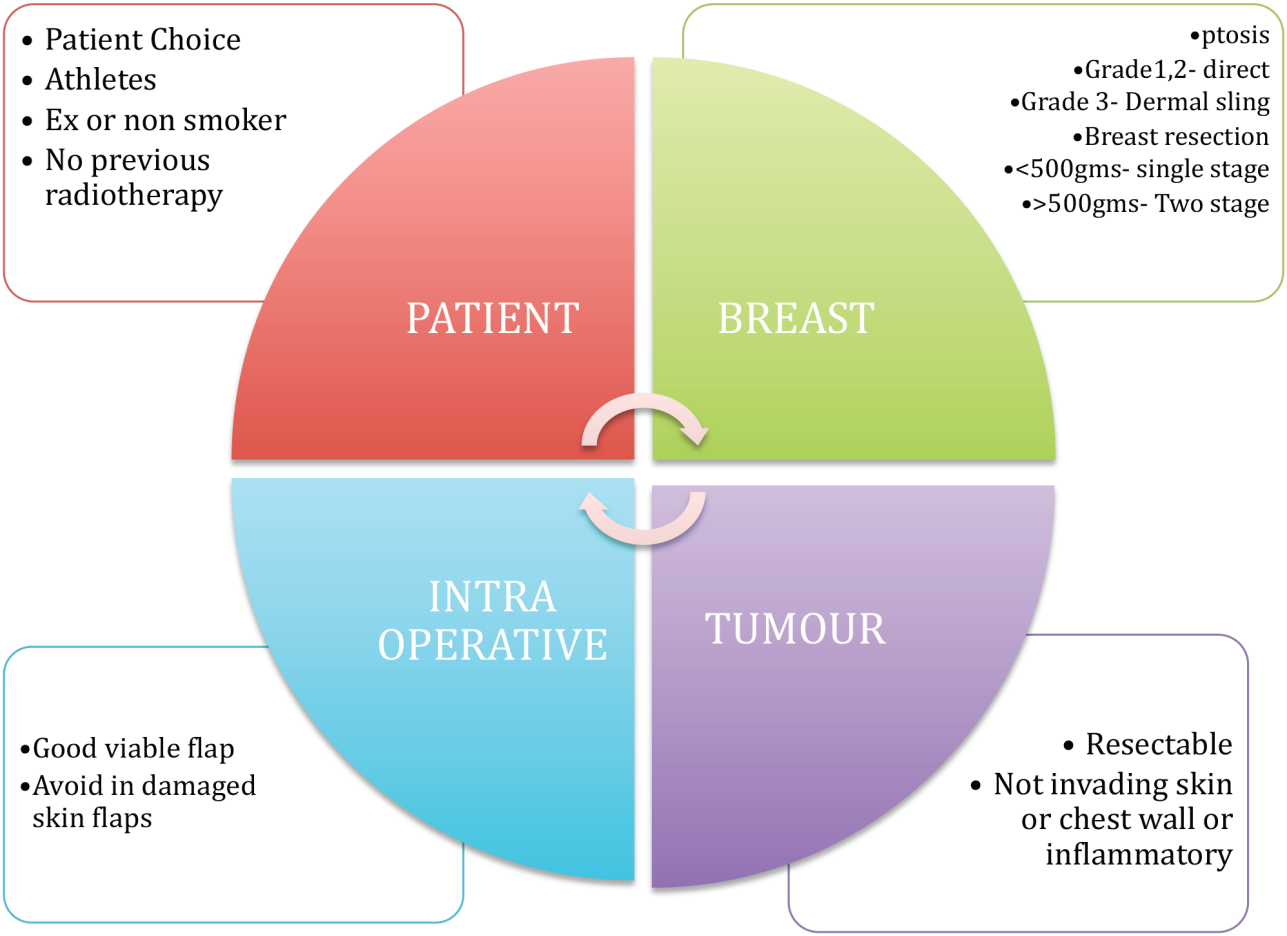
Selection criteria for prepectoral breast reconstruction.

Prepectoral implant reconstruction can be considered in anyone who would normally be considered suitable for an implant breast reconstruction. The following is a list of ideal selection criteria that are particularly appropriate in the early phase of the learning curve and shown in Figure 1.

- Patient choice.
- Patients with a reasonable subcutaneous layer over the breast tissue and options for fat grafting.
- Non- or ex-smokers.
- Well-perfused mastectomy skin flaps.
- No history of neoadjuvant radiotherapy.
- Patients with an active lifestyle, particularly athletes who require extensive use of their pectoralis muscle.
- Patients who prefer or require preserved shoulder functionality.
- Breasts with grade 1 or 2 ptosis and an estimated weight of less than 500 g.
- Breasts with grade 3 ptosis and anticipated weight more than 500 g can be offered this technique with a dermal sling.

**Figure 2.**
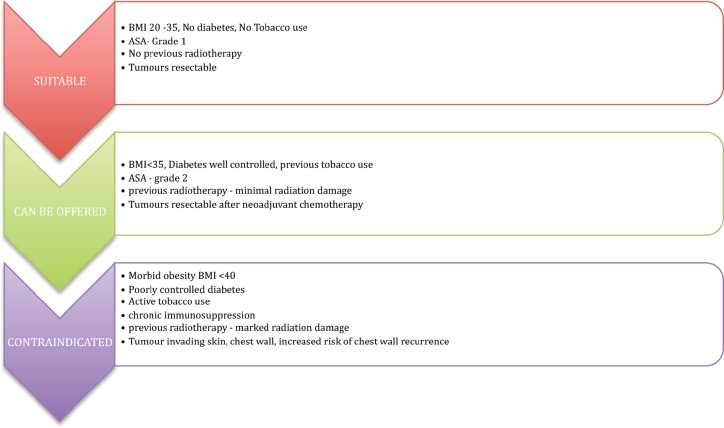
Patient selection is the most important criteria for prepectoral breast reconstruction.

Patient selection is vital, as the presence of risk factors is well known to be associated with adverse outcomes. An overview of the patient selection criteria is represented in Figure 2. The authors all consider that patient choice is of vital importance, particularly in patients such as athletes who prefer to maintain shoulder function and avoid damage to the chest wall muscle.

The procedure is offered to patients who are fit and well, with no major comorbidities or well-controlled comorbidities, body mass index (BMI) < 35, no previous radiotherapy damage and with a resectable tumour.

There is an increased risk of perioperative complications in the presence of elevated BMI (< 40), poorly controlled diabetes mellitus, immunosuppression and previous radiation damage; these conditions can be considered as relative contraindications.

The technique is avoided in tumours involving the skin, chest wall muscle, locally advanced tumours, inflammatory breast cancers and in tumours with an increased chance of chest wall recurrence.

## Technique

### Single-stage/two-stage breast reconstruction

Prepectoral implant-based reconstruction can be performed as single-stage or two-stage reconstruction of the breast. In Europe, single-stage prepectoral-based implant reconstruction is often preferred, while in the USA, two-stage reconstruction using a tissue expander is more commonly performed. However, the selection of technique would be primarily dictated by the quality of the mastectomy flaps, the risk factors and desires of the individual patient, and the need for adjuvant therapy. A two-stage reconstruction is generally regarded as safer if risk factors are identified. One advantage of two-stage is that it can include routine fat grafting at the second stage.

## Essential requisites for success of the surgery

Any type of standard skin-sparing or nipple-sparing mastectomy incision can be considered, and decisions will depend on breast form and if skin reduction is required. In general, incisions should be planned to minimise interruption of subcutaneous vasculature; those around the areola are considered high risk, and all incisions should be planned to allow double layer closure with double-breasting [5]. In one-stage nipple-sparing mastectomy, particularly success is largely dependent on the quality of the mastectomy and as such this task can only be performed by a surgeon who is expert in this procedure, with a proven minimal rate of mastectomy flap complications. Preservation of the subcutaneous layer of the mastectomy flaps and the perforators that vascularise them is the key to success of this surgery. To a degree, the thickness of the subcutaneous layer can be evaluated preoperatively using digital mammography or magnetic resonance imaging [6, 7]. A well-vascularised mastectomy flap is vital for integration and neo-vascularisation. The vascularity can be assessed intra-operatively by observation or using special devices depending on availability. The surgical plan may occasionally be required to be modified intra-operatively based upon such observations (e.g. conversion from single-stage to two-stage, with or without skin reduction).

Intra-operatively, the skin flap vascularity is the key to the success of this surgery. Most authors clinically assess flap vascularity based on colour, absence of dermal exposure and flap damage from diathermy. If devices to assess skin flap vascularity are available, the authors would advise to use it, particularly in situations when there is uncertainty about flap viability. If skin flap vascularity is compromised, other methods of reconstruction must be adopted.

## Types of implant cover

### Complete coverage

A complete cover or coverage ensures that the acelluar dermal matrix (ADM)/mesh is placed exactly in the required position with minimal fixation [8, 9]. A few studies have demonstrated a decreased incidence of capsular contracture following complete coverage [10, 11]. At present, there is only a single preshaped complete ADM coverage available in Europe although others are being launched. The remaining ADMs/meshes are available as flat sheets and need to be secured together to form one unit. The advantages of complete coverage include complete cover for the implant and ease of positioning the implant without implant displacement or rotation. However, the disadvantages include the requirement for incorporation of a large volume of mesh.

### Anterior coverage

Anterior cover or coverage results in coverage of implant anteriorly, so the posterior coverage is formed by the underlying pectoralis major muscle. The anterior coverage requires more technique and creativity to form the implant pocket, compared to complete coverage. However, it may more closely mimic the function of the pectoral muscle in implant reconstruction in reducing implant visibility along the upper pole. There may be an increased risk of implant rotation and herniation.

### Anterior coverage and dermal sling

In large ptotic breasts, a dermal flap along with the ADM/mesh can constitute a complete prepectoral pocket. The presence of a dermal flap contributes to lower pole soft tissue coverage while the ADM/mesh completes the coverage superiorly [12]. Recently, Thuman *et al* [13] demonstrated that (21 patients/37 breasts) wise pattern incision with a dermal sling is a suitable option in ptotic breasts in patients who have a high BMI. However, patients must be warned about the increased risk of perioperative complications.

## Types of meshes

A variety of ADMs and meshes are available and any that have a proven ability to integrate and provide support can be utilised. The ideal properties of meshes have been described in Table 2. Most are available as a flat sheet [14]. Some are contoured, and all the ADM’s can be fenestrated or formally meshed to increase conformability. There are different types of natural and synthetic meshes available in the market and their use is decided based on local availability, patient and surgeon preference and the cost-effectiveness.

## Cost

The cost appears to be of importance, particularly with the need for cost-effectiveness and efficient use of resources in the National Health Service and other Health Services. The availability and the cost of meshes vary across the UK, Europe and the USA. The biological meshes cost more than synthetic meshes, and most meshes are available as flat sheets, apart from Braxon, which is preshaped complete-cover porcine-derived mesh available only in the UK [13].

On average, the meshes cost was as follows.

Biological meshes cost:

- 16 cm × 8 cm piece (128 cm^2^): £1600 to £1800
- 18 cm × 10 cm piece (180 cm^2^): £2200 to £2500
- Preshaped Braxon mesh 30 cm × 20 cm (600 cm^2^): £2100
- In the US approximate cost: $3000 to $3500

Synthetic meshes are cheaper with 16 cm × 20 cm at approximately £400 (€500)

## The choice of mesh?

The choice of mesh is influenced by local availability, patient’s desire, cost-effectiveness and the expertise of the surgeon. A direct comparability and recommendation of a single product is difficult to make due to the heterogenous nature of the products and data availability to date. It is well known that biological meshes undergo collagen remodelling and revascularisation, while synthetic meshes integrate through fibrosis.

## Mesh integration

The ideal properties of a mesh are listed in Table 2. Biological meshes integrate through remodelling and neovascularisation. Synthetic meshes incorporate through fibroblastic and foreign body reaction [15]. However, all meshes need to be in intimate contact with the mastectomy flap to undergo integration. Hence, it is important to minimise seroma formation and promote wound healing [16].

## Pre/intraoperative

All patients must receive at least one dose of antibiotic at induction, and postoperative antibiotics can be administered after risk stratification or per the local hospital policy.

## Technique

The mastectomy can be performed using any preferred technique. However, it is essential to minimise diathermy or retraction injury to skin flaps. Any mastectomy for cancer should prioritise clearance over the index lesion. The mastectomy needs to be carried out in the normal breast oncological plane, ensuring as much as possible that all breast tissue is removed, but the limits of this plane need to be respected. Overlying skin only needs to be removed if a clear plane of subcutaneous tissue is not present. Preservation of pectoralis fascia enables easy fixation of implant coverage.

It is desirable to close the lateral wall from the axilla and reduce dead space inside the chest wall by quilting. A snug implant coverage is believed to reduce the rate of seroma formation.

### Complete coverage technique

Braxon is a pre-shaped (Decomed S.r.l., Venezia, Italy), 0.6-mm thick, porcine, non-cross-linked ADM. It needs to be hydrated in saline for a minimum of 10 minutes. The selected implant is placed and the mesh implant coverage is formed *ex-vivo*. The technique has been well described previously [17]. The edges of the mesh are secured with absorbable sutures (2-0 Vicryl) to form a snug pocket. The mesh implant coverage is placed over the chest wall and the unit is secured to the chest wall using three cardinal sutures at 12, 3, 9 o’clock position using 2-0 vicryl suture.

### Anterior coverage technique

Biological or synthetic meshes can be used for the anterior coverage technique. If ADM is used, two sheets of ADM are sewn together, and then incised to achieve an optimal fit over the implant. The ADM is sutured cranially to the superficial thoracic fascia and medially to the chest wall. Then the implant is inserted, and to keep it in place, lateral sutures fix the ADM to the thoracic wall and caudally the ADM is fixed to the fascia in the inframammary fold. If a synthetic mesh is used, the mesh is sutured cranially in the same way as the ADM, but covered around the implant medially, laterally and caudally without further sutures.

### AlloDerm (USA) technique

The technique has been previously described by the author in detail [18] and a summary is provided below.

The planned footprint of the reconstructed breast is marked on the chest wall. The location of the inframammary suture line is marked approximately—5 cm below that of the planned infra-mammary fold (IMF) location on the reconstructed breast. A sheet of acellular dermal matrix (AlloDerm; Allergan Corp, Irvine, California, USA.) is then placed in the breast pocket, and a suture line is first placed horizontally between ADM and the underlying pectoralis muscle fibres, approximately 3 cm above the planned IMF. The ADM is then pulled down and folded at the IMF where it will come up and over the lower pole of the tissue expander, off the chest wall. At this location, a second suture line is placed through the folded, double layer of ADM to the underlying chest wall. This ‘cuff’ of ADM at the IMF provides improved soft tissue support of the lower pole of the implant, where the ADM is then folded up and over the anterior surface of the prosthesis.

At this point, a tissue expander is placed in the breast, and the remainder of ADM is pulled up and over the entire anterior surface of the expander. The medial, superior and lateral borders of the ADM are sutured to the chest wall, at the borders of the expander. This provides full ADM coverage of the tissue expander. The lower pole ‘gutter’ of ADM, created by the enveloping of the lower portion of the implant, both anterior and posterior, allows for the reduction of the risk of future implant descent.

Next, 1 or 2 drains are then placed in each breast, and the ADM is fenestrated if necessary. Intraoperative expansion then proceeds cautiously, with care taken not to stretch the mastectomy skin flaps too aggressively. In both groups, expansion begins between 2 and 3 weeks postoperatively. For the prepectoral group, drains are left in place for a minimum of 3 weeks. After these time periods, drainage below 20 cc per day, for three straight days, will allow for drain removal.

### Composite ADM/synthetic mesh technique

The footprint of the breast is marked in the standing position with the breast supported for the upper and medial markings. After mastectomy, washout and re-prep, a contoured ADM (ARTIA; Allergan Corp, Irvine, California, USA) is thoroughly rinsed and inserted. The ADM is initially anchored at the medial IMF position. It is then anchored at the most upper medial margin of the breast footprint (pre-operatively marked), with slight tension between these two anchoring points. A third anchoring point is then created laterally, usually to the serratus fascia, again with slight tension between the anchoring points. Intervening sutures are then placed and all sutures are knotted from inside the pocket, under the ADM. The principle is that the ADM behaves as the pectoral muscle would, limiting the upper and medial projection of the implant and disguising it. The pocket is then completed with a synthetic mesh (TiLioop; PFM medical, or Tigr Mesh; Novus Scientific). This mesh is sutured around the remaining lateral and inferior margins of the footprint, forming a lateral and inferior sling to limit and support the implant. The implant is then inserted and the mesh is sutured to the free edge of the ADM, trimming any excess. One drain is inserted in the inferolateral gutter. The pocket would be formed in the same way for a 2-stage procedure and the tissue expander filled initially with air.

## Dressing and drain

Dressings are determined by local policy and surgeon preference. Negative pressure dressings can be used prophylactically to reduce wound problems although there is minimal evidence of benefit.

Closed suction drains are required and should be tunnelled well away from the implant pocket. Meticulous care of drain exit sites is necessary. The drains are usually removed when the drainage is minimal over 24 hours varying from 10 to 30 ml/day as per the local practice and surgeon preference. In our experience, this is usually 5–10 days post-surgery.

### Advantages

The advantages of the technique include; avoidance of the animation effect observed in subpectoral techniques; avoidance of chest wall muscle dissection with subsequent pain and potential effect on shoulder function [4]. Overall recovery is believed to be quicker than subpectoral reconstruction.

## Adjuvant therapy

Early experiences reveal that both adjuvant chemotherapy and radiotherapy are well tolerated with prepectoral breast reconstruction [19]. However, patients may need further interventions, including lipomodelling following postmastectomy radiotherapy. Hani *et al* recently observed that prepectoral breast reconstruction had similar outcomes in the setting of postmastectomy radiation therapy [20].

## Planned post-mastectomy radiotherapy

In the short term, our experience shows that post-mastectomy radiotherapy appears to be well tolerated in immediate prepectoral implant-based breast reconstruction with no excess adverse effects. One of the authors, Sbitany *et al* [20], recently published their experience with 14% of patients receiving postmastectomy radiotherapy and found no difference in complication rates between prepectoral and subpectoral implant-based breast reconstruction. The authors agree that planned post-mastectomy radiotherapy would be beneficial in prepectoral breast reconstructions for two reasons, including the decreased rate of capsular contracture with the use of ADM [21] as it reduces fibrosis, and secondly, it offers protective effects [22]. Hence, we recommend that prepectoral implant-based breast reconstruction can be offered to patients who need planned post-mastectomy radiotherapy.

However, we do acknowledge that the data available so far are based on case series and long-term followup is warranted to completely understand the effect of post-mastectomy radiotherapy and assess the long-term outcome, including capsular contracture and cosmesis.

The experience with previous radiotherapy and neoadjuvant radiotherapy is limited and influenced mainly by the degree of damage caused by radiotherapy and patient preference. Hence, we recommend that each patient should be individually assessed, and if suitable, the procedure can be offered provided the patient completely understands the risks, benefits and the need to have an alternative method of reconstruction if it is unsuccessful.

## Complications

### Seroma formation

Seromas will form to a degree in all cases after drain removal. Opinion varies as to the management of them. Some experts consider it essential that all are drained, either with image guidance or clinically. Some experts would manage conservatively and only aspirate if the seromas are judged to be expanding. Any seroma drainage procedure requires very strict aseptic technique and a tunnelled approach. Persistent seroma can increase the risk of wound dehiscence and prevent ADM/mesh integration.

### Red breast syndrome

This effect is rarely seen due to advancement in the manufacture of the ADMs/meshes. However, to reduce its incidence, it is essential to wash the ADM/mesh before insertion. If red breast syndrome occurs, wound infection must be ruled out but the condition can be managed conservatively although it may take several weeks to completely resolve.

### Wound dehiscence/skin necrosis/infections

Minor or superficial wound dehiscence should be aggressively managed in the clinic. Major dehiscence, flap ischemia and infections require urgent re-operation. Various techniques of implant salvage have been described and should be employed with implant removal only if these measures fail.

### Rippling

Rippling is a recognised side effect of this surgical procedure and patients must be well informed preoperatively. The reported incidence of rippling varies between 0% and 35% [23]. It is likely to be influenced by the type of implant used and the technique of implant coverage. Women with a thick subcutaneous fat layer may be considered more ideal for this procedure. In addition, some implants (with a greater percentage of gel fill) may be considered more preferable. Care is required with cohesive gel implants, where the upper lip can be visible and under-filled implants will be more likely to ripple. Patients must be informed preoperatively of this risk and the options for corrective surgery, including lipomodelling. Fat grafting is a common adjunctive procedure.

## Outcome data to be collected and audited

We recommend that all surgeons collect data regarding early and late outcomes and that all patients should have pre- and post-operative photographs. Our table below (Table 3) suggests audit standards for recordable criteria based upon the literature and our experience.

The incidence of reoperation and revision must be monitored.

It is recommended to use patient-reported outcome measures (PROMS) to assess the patient experience of information and outcomes

## Conclusion

Prepectoral or muscle-sparing implant-based breast reconstruction provides a safe and effective alternative to traditional invasive methods of breast reconstruction. It is simple and it avoids problems related to animation and shoulder dysfunction while preserving natural anatomy. However, rippling is an adverse effect associated with this technique, and patients should be informed. The technique appears to add a new dimension to implant-based breast reconstruction.

## Conflicts of interest

H Sbitany is a consultant with the Allergan Corporation. M Nahabedian is a consultant for the Allergan Corporation and Chief Surgical officer for PolarityTE (Salt Lake City, UT). Hilton Becker is a consultant for Mentor Corporation. The other authors have no conflicts of interest to declare.

## Funding statement

The authors did not receive any funding for this work.

## Figures and Tables

**Table 1.** Indications for prepectoral breast reconstruction.

Indications
Immediate breast reconstruction Immediate delayed breast reconstruction following neoadjuvant therapy Delayed breast reconstruction Risk-reducing surgery Breast revision surgery for Animation Capsular contracture Breast deformity Muscular problems associated with submuscular implant reconstruction

**Table 2.** Ideal properties of the mesh.

Minimal inflammation response
Fast integration
Easy malleability
High strength
Cost-effectiveness

**Table 3.** Outcome parameters that need to be monitored.

	Monitor	Desirable	Literature
Data	Early and late outcomes must be monitored		Essential
PROMS	Patient experience and outcomes	At 3 months and 2 years	Essential
Unplanned readmission	Preferable time within 3 months	< 10%	15%–18%
Unplanned resurgery	Preferable time within 3 months	< 5%	<18%
Implant loss	Minimum 3 months	< 10%	Up to 20%
Infection	Immediate up to 3 months	< 10%	Up to 25%
Incidence of revision must be monitored	Lipomodelling Revision surgery	< 10% < 10%	Up to 35%
